# On DNA Signatures, Their Dual-Use Potential for GMO Counterfeiting, and a Cyber-Based Security Solution

**DOI:** 10.3389/fbioe.2019.00189

**Published:** 2019-08-07

**Authors:** Siguna Mueller

**Affiliations:** Independent Researcher, Kaernten, Austria

**Keywords:** cyberbiosecurity, DNA signatures, bio-cryptanalysis, bio-cyber hacker, insecure channel, GMO counterfeiting, cryptographic applications, knowledge-based methods

## Abstract

This study investigates the role and functionality of special nucleotide sequences (“DNA signatures”) to detect the presence of an organism and to distinguish it from all others. After highlighting vulnerabilities of the prevalent DNA signature paradigm for the identification of agricultural genetically modified (GM) organisms it will be argued that these so-called signatures really are no signatures at all - when compared to the notion of traditional (handwritten) signatures and their generalizations in the modern (digital) world. It is suggested that a recent contamination event of an unauthorized GM *Bacillus subtilis* strain (Paracchini et al., 2017) in Europe could have been—or the same way could be - the consequence of exploiting gaps of prevailing DNA signatures. Moreover, a recent study (Mueller, 2019) proposes that such DNA signatures may intentionally be exploited to support the counterfeiting or even weaponization of GM organisms (GMOs). These concerns mandate a re-conceptualization of how DNA signatures need to be realized. After identifying central issues of the new vulnerabilities and overlying them with practical challenges that bio-cyber hackers would be facing, recommendations are made how DNA signatures may be enhanced. To overcome the core problem of signature transferability in bioengineered mediums, it is necessary that the identifier needs to remain secret during the entire verification process. On the other hand, however, the goal of DNA signatures is to enable public verifiability, leading to a paradoxical dilemma. It is shown that this can be addressed with ideas that underlie special cryptographic signatures, in particular those of “zero-knowledge” and “invisibility.” This means more than mere signature hiding, but relies on a knowledge-based proof and differentiation of a secret (here, as assigned to specific clones) which can be realized without explicit demonstration of that secret. A re-conceptualization of these principles can be used in form of a combined (digital and physical) method to establish confidentiality and prevent un-impersonation of the manufacturer. As a result, this helps mitigate the circulation of possibly hazardous GMO counterfeits and also addresses the situation whereby attackers try to blame producers for deliberately implanting illicit adulterations hidden within authorized GMOs.

## 1. Motivation

The cyber-physical nature of biotechnology raises unprecedented security concerns, and “Cyberbiosecurity” has been recognized as a critical imperative to “help safeguard the bioeconomy” (Murch et al., 2018; Peccoud et al., 2018). One of the critical new challenges concerns the gap between a (digital) description of a certain product and its actual (physical) realization. This was first illustrated by Peccoud et al. (2018) as they experienced major difficulties when trying to reproduce purported sequences of a plasmid sent in the mail. The actual expression characteristics of the plasmid were drastically different than what was expected from their description.

As noted by Peccoud et al. (2018), the security risks of the problems at the interface between the digital and biological/physical realms are profound. A related (but much more problematic) incident recently emerged in several countries of the European Union (Paracchini et al., 2017). Nowadays, many food and feed additives result from fermentation of genetically modified (GM) microorganisms. Microbial synthesis of vitamin B2 (riboflavin) often involves GM *Bacillus subtilis* production strains. According to European Guidelines (EFSA, 2011), for additives produced with GM microorganisms, it is necessary that in the final product neither the production strain nor its recombinant DNA can be detected. However, in September 2014, viable GM *B. subtilis* spores were detected in a consignment of vitamin B2 feed additives imported from China. Molecular characterization confirmed that these were not the strains that the manufacturers claimed to be using (Paracchini et al., 2017). In other words, the description of the product (as authorized within the EU) did not match the actual one (which was shown to harbor several unauthorized GM modifications).

The European Union has strict GMO regulations and testing mechanisms in place to determine unauthorized GMOs and to ensure compliance with regulations. The ones that are considered most reliable in fact offer real-time PCR detection of GMO-specific signatures (Permingeat et al., 2002; Levine, 2004; Allen et al., 2008), yet, herein, their role and functionality is challenged.

Originally, DNA signatures were invented to accurately distinguish between a target genome (or a set of genomes) and all other background genomes (Phillippy et al., 2007). For practical reasons, research has focused on balancing the tradeoff between signature sensitivity (the number of genomes that share the signature) and specificity (the number of genomes that do not possess the signature). With advancements in genetic engineering, however, it has become possible to actually insert artificial signatures (e.g., Gibson et al., 2010), whereby it has become possible to differentiate artificially modified from natural organisms.

DNA signatures based on integration sites between the transgene insert and the flanking DNA make use of this same idea. While these types of signatures have been the paradigm of GMO detection for decades, this article strongly challenges the function of such signatures, especially relative to intended manipulations.

Both traditionally and in the cyber-domain, signatures have long served as a valuable tool to guarantee the integrity and authenticity of the document being signed. However, the very concept of signatures in the cyber-realm first needed to be redressed as the Internet is susceptible to intrusions that are not existent in the traditional setting. Analogously, it is argued here, that unique signature vulnerabilities exist in the biologic domain.

A very recent study (Mueller, 2019) demonstrates that the existing DNA signature paradigm may be exploited via previously unrecognized forms of attack. It is suggested that new gene editing technologies can be used to create plants that are genetically modified in harmful ways, either in terms of their effect on the plant itself or in terms of harming those who would consume foods produced by that plant. This possibility opens up an unrecognized avenue for bioterrorism or biocrime—either by maliciously modifying a natural organism or (perhaps more perniciously) sabotaging a previously approved GMO. The role that DNA signatures play here is critical. The problem is not only that any clandestinely introduced manipulations are difficult to detect, but that the standard verification of DNA signatures leads to a false sense of security, as illegal or detrimental alterations can be made without changing the authenticating identifiers. This enables the adulterated product to pass as the original if only the identifiers are examined.

This article offers a detailed analysis of risk potentials of DNA signatures, with special focus on the identification of agricultural GMOs. Based on lessons learned from the cyber-domain, specific vulnerabilities are highlighted, and recommendations are made how these new risks can be mitigated.

### 1.1. Outline

Section 2 analyzes the role of DNA signatures as conceptualized in a broader framework inspired by cryptography. Section 3 describes specific risks arising in the biological realm, how conventional DNA signatures can lead to new forms of counterfeiting attacks. Section 4 considers practical issues for performing such attacks and overlays them with their cyber-based conceptualization. The combination of these two leads to specific recommendations how DNA signatures may be improved. A method how enhanced DNA can be realized through specific cryptographic tricks complemented by a suitable physical realization is described in section 5.

## 2. From Cryptographic to Biologic Signatures

This section gives the necessary background how traditional signatures were recaptured in the cyber domain, to establish analogous security features. The insights derived will help distill critical vulnerabilities in the biologic domain.

### 2.1. What Needs to be Protected

While the cyber realm is shaping our everyday lives, its underpinnings can be traced back for many centuries. Originally, it was in the form of secret exchange of messages during times of war. Out of this evolved the discipline of cryptography which later branched out into various cyber related disciplines. As cryptographic insights and ideas have been an important component of cybersecurity, it is worthwhile to analyze the underlying principles, to help guide their application for DNA signatures.

#### 2.1.1. Cryptographic Goals

According to Stinson (2005), the objective of cryptography is, “to enable two people, usually referred to as Alice and Bob, to communicate over an insecure channel in such a way that an opponent, Oscar, cannot understand what is being said.” The core issue lies in the (insecure) channel, as summarized by Claude Shannon in 1948 (see MacKay, 2003), “The fundamental problem of communication is that of reproducing at one point either exactly or approximately a message selected at another point.”

Although usually not conceptualized this way, it can be beneficial to rephrase essential cybersecurity principles in terms of insecure channels. Doing that will help filter out parallels as well as differences to the biologic domain. For instance, for the well-known CIA (or AIC) triad (see Table 1), this means:

- **Confidentiality**: The goal is to limit access to information (or, the process, and production of bioengineered products) from reaching the wrong people. Alternatively, the defining characteristics of the channel (“the message”) can be seen as “who is allowed to have access.” This “message” is intended to not change in the life-cycle of the entire information process.
- **Integrity**: This is about ensuring that information (or, a bio-manufactured entity or process) is trustworthy, consistent, and accurate over its entire life-cycle. Alternatively, in terms of a channel, “the message” is the information (content) to be secured. This should be the same at either end of the secure channel, that is, wherever and whenever it is asked for.
- **Availability**: This feature aims at guaranteeing reliable access to information (or, any cyberbiosecurity process). Alternatively, “the message” to be secured is “what is available” (to authorized individuals). This very information should be the same across different points of the channel (wherever it is needed).

**Table 1:** Cryptographic concepts and goals. In the cyber-domain, many of those can be addressed via digital signatures.

**Crypto/cyber goals**	**Description**	**Cryptographic solution**
Confidentiality	This is a service used to keep the content of information from all but those authorized to have it. Secrecy is a term synonymous with confidentiality and privacy. There are numerous approaches for providing confidentiality, ranging from physical protection (e.g., a box with a lock, a sealed envelope, or a wall-safe) to mathematical algorithms which render data unintelligible	Digital signatures, access control, hardware protection
Data integrity	This is a service which addresses the alteration of data. To ensure data integrity, one must have the ability to detect arbitrary errors, as well as manipulation by unauthorized parties. Data manipulation includes such things as insertion, deletion, and substitution	Hashing, message-authentication protocols, digital signatures
Authentication	This is a service related to identification. This function applies to both entities (e.g., a person, a credit card, an information-carrying product - including one that is biomanufactured) and information (in particular, the source of information, including its origin, date of origin, data content, time produced, etc)	Digital signatures, passwords, authentication protocols, challenge and response
Availability	Is a guarantee of reliable access (to information, computers, specific components or systems, etc) by authorized people	Updates, backups, firewalls, proxy servers, physical protection
Non-repudiation	This is a service which prevents an entity from denying previous commitments or actions. When disputes arise due to an entity denying that certain actions were taken, a means to resolve the situation is necessary	Digital signatures, public-key schemes, trapdoor functions, commitment schemes

*It is suggested that these conceptions can help identify key functionalities of biologic signatures as well*.

Although genetic engineering is not dealing with digital or electronic “communication” and “messages,” there are critical parallels - as well as differences - that are worth investigating. Traditional (noisy/insecure) communication channels are a telephone line, a flash drive, or computer network, for example. These show that communication is not exclusively understood as “information going from one *place* to another” (MacKay, 2003). When we write a file on a flash drive, “we'll read it off in the same location - but at a later time” (MacKay, 2003). MacKay gives the example of “reproducing cells, in which the daughter cells' DNA contains information from the parent cells.” This is a “noisy” channel, as this process is subject to (unintended) mutations or change.

### 2.2. Cryptographic Signatures

Arguably, if there has ever been a single paradigm that has been most influential in the security setting, then it has been that of digital signatures. (Written) signatures are providing a number of critical services, including non-repudiation, entity or data origin authentication, and identification. When electronic signatures were first developed, a redressing of the traditional concept was required. Consequently, they too, can now be used to guarantee analogous safety features in information-theoretic communication systems (see Table 2).

**Table 2:** Principles and features of digital signatures as counterparts of traditional signatures (and with the intent toward their generalization to DNA signatures).

**Digital signatures**	
How they work	“Public-key” signatures rely on the usage of specific secrets - the keys used to generate a signature. They are generated by applying a mathematical formula or an algorithm, to scramble the information into a string of digits
Who can produce a valid signature?	Only the holder of the private (secret) key–the signer–can produce such an “electronic autograph”
Who can verify a signature?	In the public-key setting, the signature can be verified by anyone
**Useful features**	
They provide authenticity and enable supply chain security	For messages distributed through a non-secure channel, a properly implemented digital signature gives the receiver reason to believe the message was sent by the claimed sender
They provide data integrity and ensure anti-counterfeiting	Any change in the message after signature will invalidate that signature, which ensures the integrity of the signed data (“the message”) against tampering or corrupting during transmission
They are binding	Once it is published, a signature cannot be altered or repudiated
What can be signed?	As with anything in the cyber-realm, the message is an alphanumeric string, including anything that can be represented as such (genomic information, producer information, processes used, etc)

### 2.3. Information Channels at the Cyber-Biological Interface

Conceptualizing security primitives in form of insecure channels has several advantages, most notably because it directs the focus to the key players that need to be secured (“signed”). When utilizing this approach, however, Table 3 shows unique challenges in the biologic field and demonstrates that the concept of traditional signatures may not be realizable.

**Table 3:** Examples of “insecure channels” in the field of cyberbiosecurity.

**Insecure channel**	**“The message” (what is to be secured)**	**Feature of a secure-able channel**	**Comment**
DNA replication: The process of passing on a parental piece of DNA to offspring	The specific DNA sequence	The DNA sequence is the same before and after replication	Numerous cellular repair mechanism turn the potentially insecure/noisy channel into one that is secured
Artificial plasmids. These are carefully designed to lead to a specific trait. Specifics of the expressed phenotype are coded in the artificial sequences	The artificial DNA cassette	The sequence information of the artificial construct is the same, regardless of the lab or environment that it is utilized by. To be “secure-able” means that this information can be traced back to its original/intended sequence	Sequencing of the plasmid allows to reveal its complete and detailed sequencing information. While this is costly and technically demanding, this shows if the channel (the sequence information encoded by the plasmid) matches the expected sequence [as e.g., can be verified by secured databases (see Peccoud et al., 2011, 2018; Wilson et al., 2012; Dunlap and Pauwels, 2017)]
Raw data, health related information, medical databases (storage of man-made information, as opposed to sequence information in living organisms)	The digital information about medical insights, health records, etc	The digital data remain unaltered (same information regardless of when and by whom it is read out), accessible only to legitimate authorities, and whenever needed	Once the information is in place, this essentially is a cyber-problem and can therefore benefit from existing cyber-related tools
Artificial DNA sequences, DNA as information storage	The message is the information to be stored in form of artificial DNA bases	As above	Need to filter out alterations due to DNA processing. Can benefit from alignment-based methods such as distance-measures (e.g., Federhen et al., 2016) and additional coding-theory and cyber-based tools to identify, correct, or minimize any errors (see e.g., Mueller et al., 2015)
Expression of a transgene via a GMO. Targeted phenotypic trait and expression levels	“The message” is the specific transgene. The channel that aims to be protected is the transgene only “The message” is the entire genome. The channel that aims to be protected is the entire organism	The transgene achieves its targeted phenotypic expression, relative to its trait, expression level, and in the context of its intended (molecular, biologic, cellular) environment	The phenotypic expression can be influenced by illicit genetic modifications outside of the transgene. If integrity is verified with respect to the transgene only, such covertly introduced modifications are not detected. They lie outside the specific channel To obtain a secure-able channel, it needs to be the case that (1) The entire genome can be sequenced, (2) The sequencing information obtained in different contexts and circumstances always lead to the exact same sequence (possibly including predictable differences within a certain range or distance)
Modern gene-edited plants and crops (see e.g., Grohmann et al., 2019)	Unclear what the message is. This is because the intended effect is based on a range of expression levels via specific biochemical pathways, which are dependent on their context and environment (here, environment is meant across the full spectrum, from molecular to gross)	The intended outcome is a spectrum of traits, depending on the specific context and environment. Here, secure-able would mean the same spectrum of phenotypic expression, as informed by different, discrete conditions in a clearly causative way	It seems much more difficult to secure a channel like this, where there is no tangible fixed, physical message that can be identified as the key information to be protected

*The key feature of insecure channels often can be formulated in terms of existing cryptographic primitives. For instance, all channels involve attributes that aim at leaving some information unaltered (integrity). Insecure channels in the cyber domain build on the salient feature that these can in fact be “secured.” In the context of integrity this would mean that the original intended information can be recaptured. In cryptography, what needs to be secured is typically called “the message.” It is important to note that this term has nothing to do with our contemporary usage of this word. Here, it describes the defining characteristics of the insecure channel. By identifying “the message” involving biological mediums it is found that many of the insecure channels are in fact “insecureable”*.

It is evident that many of the biologic insecure channels are in fact “insecureable.” That is, in contrast to the traditional/digital domain, it is often not plausible to assign a “fixed message” which is expected to have the same value across the channel. For instance, digitally, it is easy to depict the content of a certain communication, and securing the channel means that this same content can be obtained at both channel sides (sender and receiver). Finding an analog to this for living entries often is not possible, as such “messages” constitute living and flexible entities involving indels, SNPs, jumping genes, synergistic effects, or functional relationships between different forms of information and their environment.

#### 2.3.1. The Problem With Signatures in “Insecureable” Channels

A critical property of signatures is to enhance an insecure channel, and to verify that it has been “secured.” Traditionally, the signature on a legal document would serve as the means of this conclusion. In the opposite case, any alterations to the document would invalidate the signature, indicating that the channel has not been secured yet. The same is true for cryptographic signatures. If the message is “Send $ 5 to Account xyz,” then the signature that is (mathematically) computed from this “message” would be radically different to the one obtained from the message “Send $ 5,000 to Account zyx.”

In order to secure a channel it is therefore necessary to identify the underlying “message” (see Table 3), to sign it in its entirety, and to verify whether or not the intended “message” has been retrieved. In some circumstances, the same is doable for bioengineered entities. For instance, with an artificial plasmid, it would be possible to obtain the complete sequencing information (“the message”) which may be assumed to be fixed and stable. While practically this may be costly (as it would require sequencing of the entire genome) the situation represents a “secureable” channel. Indeed, the retrieved sequencing information could be compared with an authenticated sequencing information [e.g., from an appropriate database, as suggested in (Peccoud et al., 2011, 2018; Dunlap and Pauwels, 2017)]. In this regard, the latter may even assume the role of a signature for the physical entity.

**Figure 1:**
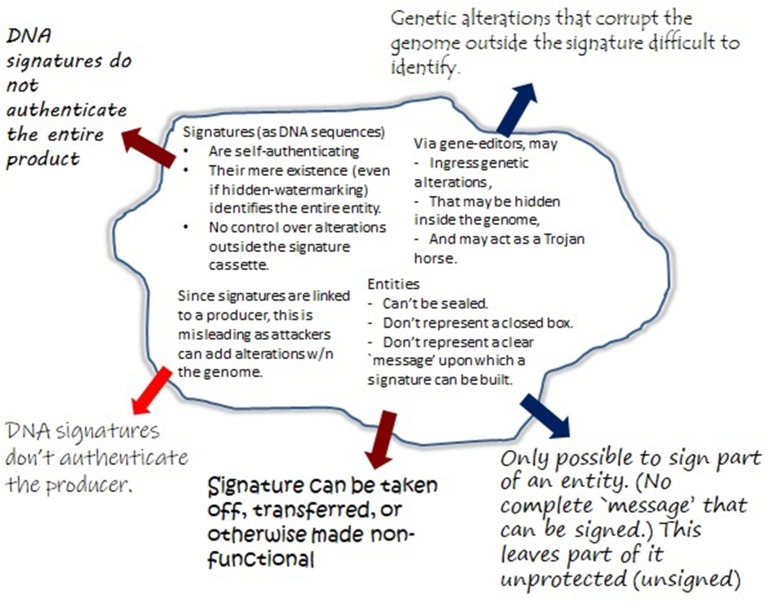
Major shortcomings of DNA signatures compared to traditional and cryptographic signatures. Traditionally, a number of security properties were obtained by sending a message concealed from outside manipulations, in form of sealed envelopes with signatures. This approach helped to ensure integrity (content of the message), its authenticity (sender and receiver), and confidentiality (the content is kept from access and alterations through unauthorized third parties). Similar features can be obtained by cryptographic signatures, by applying a mathematical algorithm (“signing”) to some fixed piece of information (“the message”). Importantly, any alterations to “the message” would not only be detected, but would invalidate the signature. The task of signing biologic entities is significantly more complex. This figure summarizes the critical vulnerabilities identified in the text (see section 3.2).

Unfortunately, GMOs (especially in the agricultural setting) cannot always effectively be sequenced in full to obtain a fixed message that remains unchanged across time and space. Here, generating a signature could be likened to signing a blank check, or worse. Figure 1 provides a “bio-cryptanalytical” summary of the main challenges that arise in this context.

## 3. The Potential and Risk of Large-Scale Intrusions

### 3.1. Accidental GM Contamination and Signature Theft for Cost-Saving Purposes

The detection of the unauthorized GM *B. subtilis strain* in Europe has led to rigorous investigations to identify the unknown genetic insertions/deletions that are responsible for the significant overproduction of vitamin B2 (Barbau-Piednoir et al., 2015; Paracchini et al., 2017). The analysis by Paracchini et al. (2017) revealed genetic adulterations in form of specific indels as well as extra-chromosomal recombinant plasmids that are presumably conferring antibiotic resistance for selection purposes and stable riboflavin expression during fermentation. Correspondence with the manufacturers revealed they were relying on known GM-strains, which means that at some point some type of identification process must have been in place. The problem arose when these authentic identifiers falsely got associated with the modified strains. The same, however, could be done by bio-cyber hackers in form of intended DNA signature misuse (theft) to masquerade an unauthorized product.

### 3.2. DNA Signature Theft With a Malicious Intent

#### 3.2.1. Signature Theft to Harm the Reputation of the Manufacturer

In the B2 contamination event, rigorous investigations between European and Chinese competent authorities led to the conclusion that the “the production strain must have been contaminated or switched before or during production” and that it concerned an “exceptional” and “singular” case (Paracchini et al., 2017). The genetic alterations turned the feed additive into something that is unauthorized in Europe. One can imagine that when done on purpose, such types of attacks may be performed with the explicit intent to harm the reputation of the manufacturer. Similarly, counterfeiters may try to ingress more harmful manipulations just to blame the producer. It is important to note that such attacks may not easily be detected, especially when nobody is looking for such (unknown) alterations. Nonetheless, even the advanced PCR methods developed by Paracchini et al. (2017) would only identify the specific unauthorized strains disclosed in Europe, but would be of no help in the detection of any modifications that have been introduced in clandestine.

#### 3.2.2. DNA Signatures for the Identification of GMOs

The core vulnerability with DNA signatures is that the mere presence of such signatures is no safeguard against alterations outside the signature cassette. In fact, the very presence of identifiers may establish an effective way to support the hostile usage of GMOs. In Mueller (2019) it is suggested that DNA signatures may intentionally be exploited to enable the counterfeiting of GM-plants that clandestinely have been genetically manipulated to harbor a hazard or other illicit trait. This may involve rather harmless modifications resulting in GMOs authorized in one jurisdiction but not in another. It could also involve much more serious forms of adulterations that turn plants into potential attack vectors, such as the intended deletion or silencing of genes or mechanisms to corrupt the various defense mechanisms employed by plants or to interfere with transgene expression levels in specific plant tissue (see also **Figure 3** below).

Such hazardous GMOs^1^ would still bear the unique DNA signatures and thereby give a false sense of security when only these identifiers are tested. Such tests would not reveal the covertly introduced modifications. Unless rigorously analyzed, either through WGS or via phenotype expression patterns, the clandestine manipulation would remain hidden. As the identification of GMOs (especially when dealing with unauthorized ones), is known as practically both difficult and costly (see e.g., Holst-Jensen et al., 2012; Arulandhu et al., 2016; Grohmann et al., 2019), this may further help evil-doers to circulate manipulated products masqueraded as the real thing.

### 3.3. The Challenge of Authenticating Bioengineered Entities

#### 3.3.1. Issues With Watermarking in Biological Mediums

The problem with DNA signatures is not only illicit adulterations that leave the signature itself unaffected. Of greater concern is the fact *that* with agricultural GMOs those signatures are stably integrated into the genome. Counterfeiters could utilize this very fact even when the signature string itself is hidden (as it is in watermarking). Modifications that potentially result in a hazardous GMO could lead to serious concerns relating to ownership and attribution especially when the manipulated GMO contains an authentic signature.

Traditionally, watermarking has been an effective means to validate ownership of a certain product. The approach has many useful applications and was utilized, e.g., by Gibson et al. (2010) for the identification of the first artificial bacterium. The incorporation of specific markers into the genome served to identify it as artificially constructed by these researchers. It is important to note that the goal was not to hide a secret. The “hiding” of the identifiers—as with other DNA watermarking methods—was for biocompatibility purposes (i.e., to not interfere with cellular processes); it was not to conceal the existence of these strings from potential signature thieves.

The problem with watermarks for identification purposes is that attackers could potentially misuse their mere presence in form of counterfeiting attacks. All they need to know is *that* a certain GMO is carrying a unique signature (or tag). As long as that sequence is used for identification, this gives a basis for attackers to ingress modification and distribute the adulterated as a counterfeit of the authentic product.

### 3.4. Signature Transferability Enables Counterfeiting

The above vulnerabilities can be summarized as, (1) the genome carrying the identifier could be corrupted at some other loci, (2) the identifiers themselves could be transferred unto an unauthorized entity, or (3) they could be duplicated (stolen), to masquerade an adulterated product as the original. Thereby, the manipulated GMO:

- Is assumed to come from a claimed authentic producer,
- And thus is believed to resemble the claimed product.

**Figure 2:**
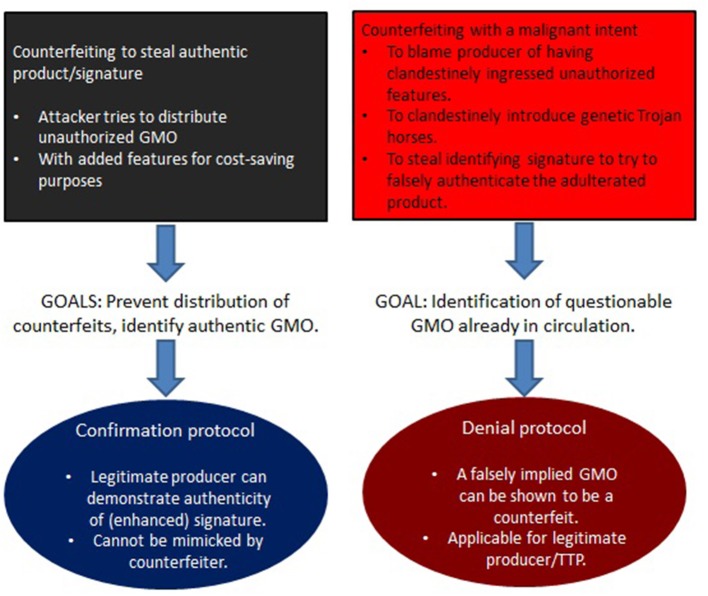
Herein, unrecognized risks involving counterfeiting attacks are identified that rely on the intentional misuse of prevailing DNA signatures (section 3). Although no such GMO counterfeit is confirmed in circulation, a recent B2 contamination event in Europe (Paracchini et al., 2017) demonstrates that these risks need to be taken seriously. Depending on the type of risk, different strategies need to be pursued. Steps toward realizing these goals are described in sections 4, 5.

It is seen that this constitutes a novel form of counterfeiting in essentially two ways. The first is to engineer a product that is cheaper to produce, cultivate, or select for (such as through the unauthorized use of antibiotic markers). Of greater concern is counterfeiting with a malicious intent, when trying to falsely attribute an adulterated product to a legitimate manufacturer. These two situations need to be addressed differently. In the former case it is paramount to keep counterfeiters from introducing manipulated GMOs (which could contain hazardous modifications) into the market. The latter concerns adulterated GMOs that attackers have already managed to bring into circulation, and that require adequate methods to assign attribution. A summary of this, along with critical requirements for a solution, is depicted in Figure 2.

## 4. Practical Considerations and Preliminary Recommendations

### 4.1. Cryptographic Primitives in the Context of Bioengineering

Cyber-security risks are often illustrated in form of a network depicting the insecure channels between users Alice and Bob. For instance, if Alice is sending an email to Bob, then this is a complex process, whereby the email message is broken down into parts, processed at various points in this communication system, until it eventually reaches Bob. The individual steps are all computers or processes, and notably, each of these can become the source of attack.

However, in the cyberbiosecurity realm, this picture is radically different. If we consider the production of a GMO, then the individual steps between this (honest) intent (or, alternatively, the goal for the willful alteration of a GMO) to the final product on the market also consists of numerous steps. Yet, in sharp contrast to the cyber-realm, here it is not the case that each and any individual node in the network is more or less equally equipped for introducing the same degree of harm.

Although the - minimal - requirements that are needed by bio-cyber hackers to misuse modern gene-editors such as CRISPR/Cas have been highlighted by many (see e.g., DiEuliis and Giordano, 2017; Dunlap and Pauwels, 2017), this does not address the feasibility and likelihood of performing attacks on entire GMOs - to the extent that these would have a noticeable phenotypic effect at a large scale.

To achieve a large-scale hazardous effect, more is required than just manipulating a few cells of a target organisms (which may even be within a secured physical environment). This leads to considerable operational, research, and manufacturing challenges. It is seen that the most critical factor lies in the actual abilities of attackers, to generate and distribute their fabricated GMOs, while evading existing screening and safety checks. It is suggested here that this establishes a scale of attack feasibility that needs to be overlaid with the previous (cryptography based) factors, to estimate the practical likelihood of intrusions.

**Figure 3:**
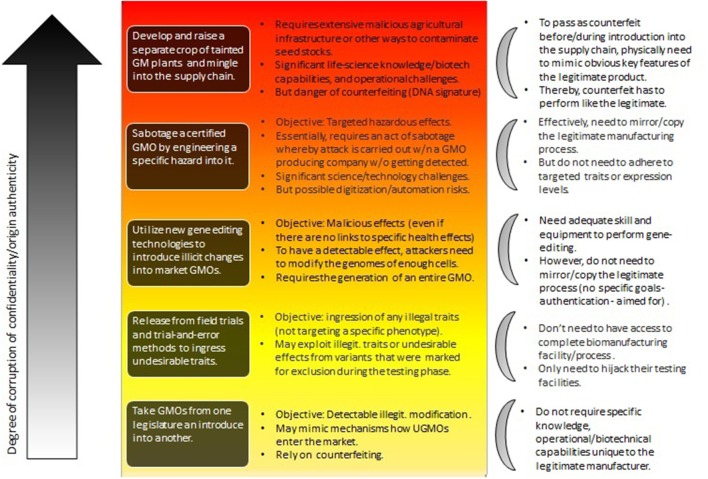
The types of attacks involving GM plants as considered by Mueller (2019) (central part of the figure), roughly ordered from bottom to top relative to their risk-potential. Their impact is also hierarchical with risks at the lower level inherited at higher-levels. Herein, the focus is on the degree to which confidentiality and authenticity are violated (see section 4.2).

Figure 3 describes such a hierarchy in the context of trying to weaponize agricultural GMOs (see Mueller, 2019).

### 4.2. Key Approach

What Figure 3 (with special focus on GM plants Mueller, 2019) shows, is the difference of the influence of the various cryptographic primitives on various levels of attack. At the lower end of the hierarchy we find attackers that can do simple gene-edits, but that don't rely on the manufacturing facility itself to produce these changes. In contrast, the top describes attackers that are able to essentially sabotage an entire GMO, or even an entire GMO production facility.

As section 2.3.1 illustrates, many cyberbiosecurity channels based on the defining characteristics of integrity are in fact insecureable. On the other hand, section 3.2 describes critical vulnerabilities in form of counterfeiting attacks. The latter are based on compromising the product itself (corrupting integrity) as well as authenticity (who generated the product). Although restoring integrity seems to be difficult (if not impossible, see above), it may be possible to strengthen channels based on authenticity and confidentiality. Indeed, as Figure 3 shows, it is the scale of compromising these (involving both digital and physical/biological entities) that determines the severity of attacks (see also Figure 1, where red arrows are associated with authenticity/confidentiality, and blue with integrity).

This suggests the following approach to help mitigate the risks associated with DNA signatures as described above. Essentially, the goal is to keep the product (especially, the signature) from all those intended to have it. This begins with the producer and proceeds along the supply chain. While several approaches have been suggested by Frazar et al. (2017) to help secure the synthetic biology supply chain, here an enhanced concept of DNA signatures is suggested to help support this, as follows. Such signatures should:

- Incorporate a strengthened feature to enable authentication of the producer. As continuous quality control testing of the production process and product purity is required to exclude contaminations and/or impurities (Hermann and Schurter, 1995; Paracchini et al., 2017), ensuring the origin of the GMO (authenticity) is most critical to secure integrity of the released product.
- Ensure confidentiality–of the enhanced signature (to prevent signature theft and transferability). That is, keep the identifier (signature) from all but those authorized to have it.
- Allow public verifiability of GMOs for the verification of authentic products.

These last two items seem to be conflicting. Yet, it will be shown below that they can be reconciled by realizing DNA signatures in two parts, one digital, and one physical.

## 5. A Cryptography-Based Method to Enhance DNA Signatures

### 5.1. Intuitive Description of Key Features

The presented solution relies on a cryptographic mechanism which had been constructed to address a challenge that arose in a completely different context. An undesirable characteristic of digital signatures is that anyone who has access to the deciphering key (in the “public key” setting this would be everyone) would be able to verify the validity of a purported signature string. This universal verifiability (or self-authentication) would be unacceptable when sensitive or private information is involved. A typical example is described by Xia (2013), when software vendors might want to sign on their products to provide authenticity to their paying customers. At the same time, however, they do not want those who have illegally duplicated their software to verify the validity of the product by being able to verify their signature.

This situation is similar to the one described above (section 3.2). DNA-signatures have been constructed so that they can be publicly verified. While this is a critical factor to support the identification of GMOs, this may also enable their potential misuse. An attacker can introduce changes into the genome without affecting the authenticating signature identifiers, allowing the adulterated product to pass as the real thing. This is of particular concern if only the signatures are used for identification. They would indeed be universally verifiable, but would not say anything about any hidden adulterations within the genome.

**Figure 4:**
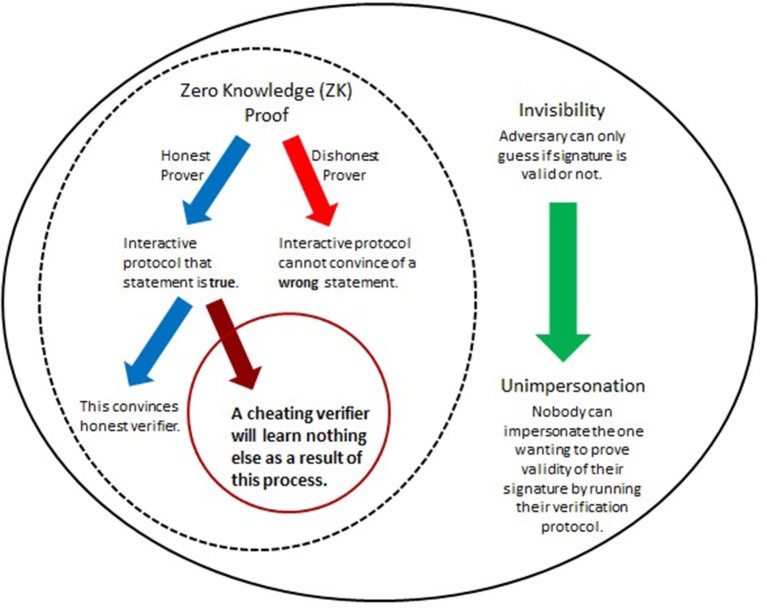
Herein, improvements of DNA signatures are obtained by utilizing cryptographic tricks that have proven useful for special cryptographic applications such as identification protocols and enhanced signatures (Menezes et al., 1996; Camenisch and Michels, 2000; Ateniese, 2004; El Aimani, 2009; Xia, 2013). At the core are (mathematical) interactive proof systems to demonstrate the (in)validity of a certain statement such as, “This is my personal PIN.” The significance of Zero-Knowledge (ZK) proofs lies in the fact that such systems can convince of the correctness of the statement without needing the involved parties to expose any details, such as, specifics of the PIN itself. ZK protocols can be overlaid with a feature that ensures authenticity of the originator of the statement or signature. When combined, this gives a powerful method to verify signatures while at the same time preventing their transferability or misuse by unauthorized parties.

The predicament of self-authenticity of digital signatures motivated the introduction of undeniable signatures (Chaum and Van Antwerpen, 1990), designated confirmer signatures (Chaum, 1995; Camenisch and Michels, 2000), and improvements (see e.g., Camenisch and Michels, 2000; Ateniese, 2004; El Aimani, 2009; Xia, 2013). Essentially, their realization hinges upon two main features as summarized in Figure 4. The first involves Zero-Knowledge (ZN) proofs of knowledge which allow (mathematical) demonstration of knowledge of the signer's secret identifier without ever having to expose this secret. The second is called “invisibility” (Galbraith and Mao, 2003) and in this context means that outsiders would not be able to distinguish between two types of secrets. This concept is critical for ensuring a form of authenticity (unimpersonation, see Figure 4 and Xia, 2013) and will be incorporated below in two ways. When invisibility is combined with ZK (Figure 4), then this also supports confidentiality - the secret is kept from all but those authorized to have it. This cryptographic framework, when applied to enhance DNA signatures, would address both of the key goals identified above (section 4.2). The involvement of the most critical elements is detailed in Figure 4.

### 5.2. Summary of the Enhanced DNA Signature Method

Designated confirmer signatures and ZK proofs were first suggested in Mueller (2014); Mueller et al. (2016) as a basis to mitigate the problem of DNA signature transferability described above. These authors also provided an explicit description of algorithms and a specific watermarking protocol to hide a representation of the digital signature component within the GMO itself.

As the underlying cryptographic framework has been summarized and enhanced by Xia (2013), this now allows for a more direct description of such DNA signatures, that allows additional improvements relative to Mueller et al. (2016). Thereby, enhanced DNA signatures can be based on two legs (via a cryptographic/digital protocol, and via DNA bases/physically), with the following main features.

- Signature strings are not self-authenticating in the sense that they can directly be verified (simply by their physical or electronic existence).
- Instead, signature verification is firstly done via a cryptographic process as summarized in section 5.1 and explained more fully in section 5.3. Thus, it is the outcome of that process that determines the conclusion, not the digital (or physical) signature sequence itself.
- Secondly, a “cryptographic fingerprint” (hash) of the cryptographic signature is converted into DNA bases via some watermarking protocol and incorporated into the genome (see section 5.4 for details).
  - In case of confirmation of a GMO (Figure 2), the presence of these DNA sequences can be publicly verified by standard hybridization methods (This part in itself incorporates no security components and serves only to tie the digital to the physical component).
  - To achieve denial of a signature (Figure 2), the above is complemented by a physical invisibility feature (Figure 4 and section 5.1). In case of dispute, when denial is required, this step can be performed by competent authorities in combination with WGS.

**Figure 5:**
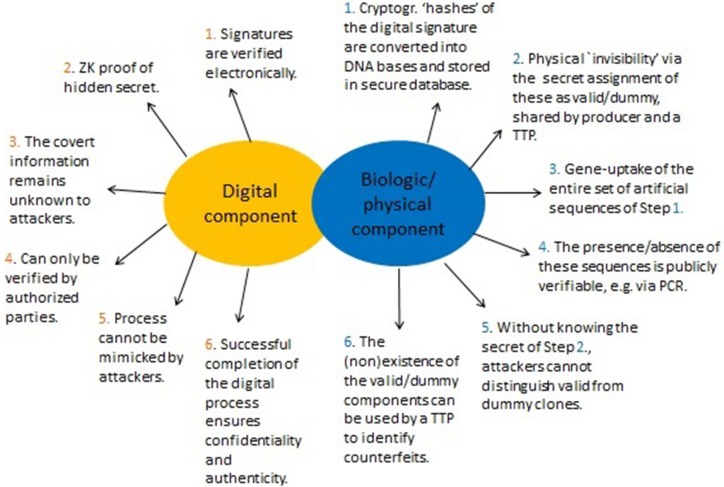
Summary of the proposed method to enhance DNA signatures. Signatures are represented and verified in two ways. One is digital and based on specific cryptographic signatures (section 5.1) by utilizing enhanced Zero-Knowledge (ZK) proofs of knowledge via a cryptographic “invisibility” property (Figure 4). The second part ties the actual (physical) GMO to the digital part and adds a physical “invisibility” feature. Consequently, it is possible to (1) Demonstrate genuineness of a legitimate signature (this can be done both physically and digitally), (2) Prevent counterfeiters from selling manipulated GMOs, and (3) Allows authentic producers to demonstrate that a falsely attributed (fabricated) GMO is not theirs. This step may require WGS and can only be performed by a TTP or competent enforcement authorities who can verify the secret assignment into “valid” or “dummy”.

For the digital part, the objective of the signer (the producer of a GMO) is to convince a buyer (e.g., key importers) of the validity of the cryptographic signature (as described in sections 5.1, 5.3). Yet, the signature string itself is not exposed during this process. Its involvement is implicitly, that is, in a hidden manner (ZK property, Figure 4). This allows it to remain concealed throughout. The physical part is primarily used for linking the digital with the actual product, but also serves to solve dispute. A summary of the method is given in Figure 5.

### 5.3. The Digital/Cryptographic Part

This section summarizes the security properties of the digital part in the framework developed above. For an explicit formulation via specific cryptographic algorithms, parameters and keys involved, see (Mueller, 2014; Mueller et al., 2016). The most important components to enhance DNA signatures are the following:

- The cryptographic verification process can be run by legitimate parties (most notably, the manufacturer and the first point of sale, Golan et al. (2004) in form of a series of check routines. The completion of these interactions establishes a mathematical proof which convinces the verifier about the (in)validity of the purported signature, including its alleged originator (authenticity).
- Nobody can see the validity of a signature without the verification protocol. In other words, an adversary who has access to a purported signature string has no choice other than a random guess to learn if that signature is valid or not (invisibility).
- The conclusion whether the digital string represents a valid signature or not cannot be misused by adversaries (who might be masquerading as verifiers) to impersonate the producer (signature theft). The argument for this is simple. If an adversary can impersonate the legitimate producer (of the signature) by successfully convincing any verifier thereof, then it must be able to run the interactive protocol. However, if an attacker can run the protocol in such a manner to convince a verifier, it must trivially know the signature's validity. In other words, if an attacker can break unimpersonation, it can also break invisibility (Figure 4).
- Even if the interactive protocol has successfully convinced the interacting parties of the (in)validity of a signature, attackers (masquerading as verifiers) cannot use any of the insights learned from the underlying mathematical procedures to demonstrate this same fact to anyone else (ZK property).

Consequently, attackers trying to masquerade a legitimate GMO cannot conduct the necessary digital confirmation protocol (Figure 2), which will make it impossible to sell their counterfeits. The important point is that only those in possession of a the secret (as required in the cryptographic protocol) are able to complete this process. Thereby, only the legitimate manufacturer of the authorized GMO can provide a digital proof of their genuineness. This step is meant to prevent introduction of unauthorized (and possibly hazardous) GMOs into the supply chain at a larger scale (see Figure 3).

The digital part gives assurance that the GMO in question was indeed produced by the legitimate manufacturers, and that it is not a manipulated product of unauthorized origin. While digital verification in many regards may be easier than physical, it needs to be stressed that this part only gives assurance about the purported GMO. Clearly, there needs to be a link to tie the above to the real, physical entity in question. This accomplished by the second leg of the protocol (section 5.4 below).

**Figure 6:**
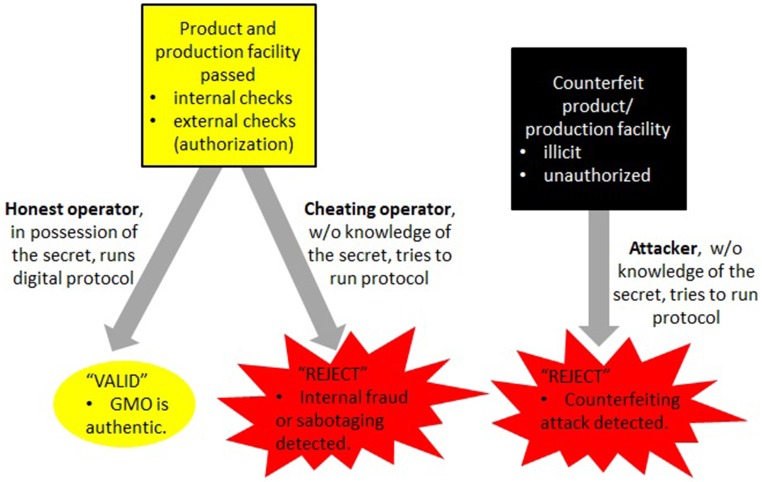
The digital part of the enhanced DNA signature method utilizes special cryptographic signatures (sections 5.1, 5.3) whereby signature verification is accomplished via a protocol rather than verification of presence or absence of a certain sequence. This gives a high degree of security and can only be achieved by legitimate producers (or their proxies) who know the underlying secret used for computing these cryptographic signatures. Attackers are not able to mimic this process and therefore cannot distribute counterfeits of GMOs by trying to masquerade them as the original product (Figure 2).

Overall, the cryptographic component ensures confidentiality and authenticity and thereby helps to prevent the unauthorized distribution of counterfeits as well as the identification of false signatures in case of concern or dispute. Assuming that the production company has verified the authenticity of the final product before its distribution, this guarantees a high degree of security of DNA signatures. See Figure 6 for a summary of this part.

### 5.4. The Physical Component—Tying the Cryptographic Part to the Actual GMO

To link the digital part with the actual GMO, it is necessary to incorporate a representation of it within the genome itself. The strong security properties of the above digital part are depicted in Figure 4. Ideally, one might try to extend these attributes to the physical domain (the actual GMO in question). A quick reflection immediately reveals major challenges. Whenever a purported DNA sequence is verified physically, e.g., via hybridization methods, then this obviously reveals the presence or absence of this sequence in the GMO, making it impossible to achieve a ZK property (the solid circle in Figure 4). This section offers some suggestions to reclaim related security features nonetheless.

#### 5.4.1. Construction of the Physical Signature Component

A standard characteristic of cryptographic signatures is that their representation in the electronic signature space looks like a random string with equal occurrences of 0′*s* and 1′*s*. However, the nucleotides within the genome do not represent an equidistribution of A,T, C and G. In Mueller et al. (2016) a method is described how the cryptographic signature can be converted into the DNA alphabet so that it is indistinguishable from endogenous DNA after insertion into the genome.

The watermarking protocol that was developed in Mueller (2014) and Mueller et al. (2016) for this purpose takes advantage of the equiprobable distribution of the cryptographic (usually, binary) alphabet, to represent binary text triplets according to the codon bias of the host genome (Figure 3 in Mueller et al., 2016). Doing this effectively camouflages the resulting DNA string so that an adversary cannot easily identify its presence within the genome (other than through WGS). In Mueller et al. (2016) this physical signature is only required in case of conflict and normally the signature remains hidden.

This approach can be enhanced, in two ways. The first is to shorten the DNA signature, so that in place of the rather lengthy cryptographic signature, only a cryptographic fingerprint (hash) (see e.g., Menezes et al., 1996) is incorporated into the genome. Such hashes have the beneficial property that they are much shorter (a few hundred bases). Yet, in practice this is sufficient, as it is infeasible to retrieve any useful information about the signature that the hash was computed from. The second improvement will make the absence or presence of DNA signature sequences publicly verifiable (section 4.2), and thereby enable identification of authentic GMOs in general circumstances, and not only during dispute.

To accomplish this, a form of “invisibility” property (Figure 4) will be achieved within the DNA signature part itself, as follows.

First, several different hashes are created from the given cryptographic signature and stored in a secured and publicly accessible database. In a second step, these are secretly assigned different values, denoted “valid” and “dummy.” This assignment of which string is of which type is secretly shared between the producer and a Trusted Third Party (TTP). The entire set of the hashes are then converted into DNA bases as in Mueller et al. (2016) and embedded into various clones.

It is important to note that since the signature components within the genome (that is, the individual hashes) are meant to be publicly verifiable, that here the watermarking protocol is not employed to hide the sequences from adversaries, but mainly for biocompatibility and practical purposes. This step may be complemented by various techniques to support the uptake of artificial sequences, relative to GC content, codon bias, repetitive sequences etc. Practical steps how the identification of the individual target sequences (e.g., through hybridization) via suitable primers can be aligned with related methods have been described elsewhere (e.g., Paracchini et al., 2017).

#### 5.4.2. Detection of an Authentic GMO (Confirmation)

Because the secret lies in the designation of the various clones as valid or dummy, the individual DNA signature hashes (without this assignment) can now serve just as previous DNA signature sequences. As artificially created constructs (hashes of the cryptographic signature), it is unlikely that these overlap with endogenous sequences of the GMO (although this could be verified a priori). As a result, their presence or absence not only authenticates a unique GMO, but also establishes a verifiable link to the cryptographic protocol (which holds the core of the security qualities of the entire protocol).

#### 5.4.3. Confirmation of a Counterfeit (Denial of an Unauthorized GMO)

The secret association of which clones are carrying valid and which mere dummy elements can also help resolve the following vulnerability not addressed in Mueller et al. (2016). Suppose an adversary (masquerading as an honest buyer) interacts with the manufacturer or their proxy in the cryptographic protocol, but manages to adulterate the authenticated GMO (see Figure 3 for practical difficulties). Rather than trying to steal a product, an attacker could try to blame a producer for creating such (possibly harmful) modified GMOs, and could try to support their claim through the cryptographic protocol associated with the verification of an authentic GMO (Figure 2).

Although the cryptographic protocol can be used by honest parties to digitally “deny” a falsely attributed signature as theirs, in the present context this is absurd. What is at stake here is not whether or not GMO manufacturers can mathematically prove that a cryptographic signature is not theirs, but that the actual (manipulated) GMO was *not* manufactured by them. This can be achieved through the secret assignment of the individual clones as valid or dummy (physical invisibility). Thus, as in the digital part, without knowledge of their secret underlying meaning (as valid or dummy), outsiders can only speculate which clone is of which type. The above vulnerability can now be resolved by a TTP or competent authority according to the following rules.

- Legitimate GMOs can be identified if the collection of clones contain the entire set of signature hashes. A collection of clones that does not include the full set of signatures is unauthentic and possibly the result of an attack.
- By definition, signature verification only involves clones carrying valid signature components. That is, as legitimate participants can distinguish the two types of clones (associated to “valid” or “dummy”) it is possible to select only those samples with the former type of signatures.
- Also by agreement, any genetic modifications (used by the attacker to blame the manufacturer, or identified via WGS) found on “dummy” clones are declared counterfeits.

The reasoning for this is that legitimate manufacturers will not introduce genetic modifications other than those they are seeking (or owning) authorization for. Thus, they will not include unauthorized modifications to any of their clones. Any additional alterations, e.g., for testing purposes, can be forced upon valid clones only, which however requires knowledge of this secret (see also section 5.4.4 below).

One of the critical goals of microbial forensics lies in the identification of the causative agent or source of a disease outbreak (Murch, 2015). This is equally important with illicit or compromised GMOs. The manipulated and masqueraded product could be mingled into legitimate supply chain and widely distributed. In theory, it seems impossible to avoid such intrusions, at least on a local level. Yet, as with microbial forensics, “resolving with high confidence whether or not the outbreak” - or the occurrence of illicit or manipulated GMOs - “manifested as a result of a natural, accidental, or deliberate event is crucial” (Murch, 2015). While more difficult to realize, the above denial part may effectively complement previous efforts to assign molecular attribution (see also Minogue et al., 2019).

#### 5.4.4. Summary and Extensions of the Denial Part

When the enhanced cryptographic signatures based on ZK proofs were first invented (Chaum and Van Antwerpen, 1990; Chaum, 1995), they were realized via very simple mathematical algorithms such as simple modular arithmetic (Menezes et al., 1996). It quickly became apparent that verifying a secret via a proof of knowledge is considerably more difficult to realize in the case of denial. Obviously, it would not be enough to just claim to not know the secret. Cryptographic realizations to support a denial feature have required somewhat more sophisticated mathematical algorithms, which may explain why these types of signatures have not received much attention.

Importantly, this same challenge of denial can effectively be extended into the physical realm. This is the essence of the denial protocol described above. Here, the role of the secret is assumed by the value of the clones (valid or dummy). Now, in form of a physical test, the challenge of demonstrating knowledge (or its opposite in form of denial) can easily be realized through hybridization methods that verify the presence or absence of the required sequences.

**Figure 7:**
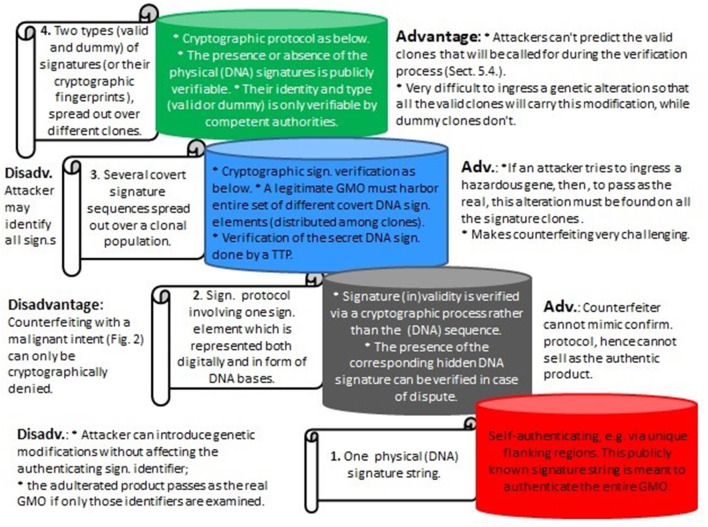
Various types of DNA signatures as considered herein, from bottom to top with increasing levels of security. 1. Represents the existing DNA signature paradigm (e.g., Levine, 2004); 2. and 3. are described in Mueller et al. (2016), and 4. (section 5) is an extension of the cryptographic invisibility feature which is central to the underlying cryptographic part in Mueller et al. (2016). (Sign, Signature; Adv, Advantage; Disadv, Disadvantage; Confirm, Confirmation).

Assuming that the artificial signatures are stably integrated into the genome, the absence of some “valid” clones will immediately point to an attack. However, much more problematic is the situation where skilled attackers could aim to circumvent that. With modern gene editors it might be possible to illegally ingress genetic hazards into the complete set of all the (different) authentic clones. As a result, the corrupted set of GMOs would carry the required set of signature elements, which could lead to critical problems concerning legal ownership and attribution. It is for reasons like this that the proposed method requires not only different signature clones, but also the added invisibility component. See Figure 7 for the different signature types considered herein and their advantages and disadvantages.

The denial part is the only part in the proposed method that relies on WGS (unless knowledge and assumed functionality of the adulterations are already part of the attack). This is necessary to identify all genetic modifications that clandestinely have been inserted into the GMO. While this is costly, WGS continues to become more practical and efficient (see e.g., Arulandhu et al., 2016). Moreover, the search could be enhanced by biochemical identification systems as was demonstrated by Paracchini et al. (2017) in the characterization of the unauthorized GM *B. subtilis* strain.

Based on their in-depth analysis, these authors came to the conclusion that some of these alterations were intentionally introduced. Nonetheless, at this point one can only speculate whether such manipulations were done as part of an actual criminal act (Figure 2). The denial component above would give authorities a method to demonstrate such types of counterfeiting attacks. Since legally authorized parties can obtain the secret designation of the type of the clones (as valid or dummy), it is possible for them to amplify and select only the valid clones and verify the presence/absence of the genetic alteration on the individual clones. As any illegal modification after approval of the authorized GMO will affect large proportions of clones, inadvertently some of these alterations will land on clones that were labeled as “dummy.” This would officially confirm that the alteration is a form of attack.

However, not all attacks need to involve genetic modifications (see Figure 3). Additional known risks involve the giving away of GMOs from laboratory or field trials. Of special concern here are trial-and-error methods (e.g., Rodŕıguez-Leal et al., 2017), whereby inside-attackers can take seeds or products exhibiting undesirable (“the error”) characteristics.

Here, the secret classification of the various clones can be helpful in the following way. Laboratories seeking to run a certain experiment can exchange the secret identifier information with a trusted party or certification authority. Thereby, they would agree to perform the trial-and-error methods only on a certain subset of the different clones. Insider attackers that are injecting “error” samples into the food/supply chain would inadvertently select from this same specific set of clones, demonstrating that the illicit sample needed to have originated from their lab, thus narrowing down possible suspects - and perhaps discouraging insiders to perform such types of illegal actions.

### 5.5. Summary of the Overall Protocol

An overall summary of the enhanced DNA signature method is given in Figure 5. It is seen that by incorporating the fundamental steps of special ZK based cryptographic signatures (Figure 4), it is possible to obtain the necessary safeguarding components as identified in section 4.2.

The strength of the described method lies in its robust identification of GMOs which cannot be mimicked by counterfeiters. For practical purposes, this may indeed be one of the most important steps to guarantee the circulation of authentic GMOs. The opposite (more costly) part of denial is practically of less concern and in fact is already minimized when authenticity and confidentiality of GMOs are ensured. Table 4 gives a survey of these opposing goals and the components of the solution as realized herein.

**Table 4:** Summary of the proposed method to enhance DNA signatures, relative to the two main goals of disputing a falsely alleged GMO or confirming a genuine one (see Figure 2).

**Performance**	**Meaning and significance**	**As realized in the proposed protocol**
True positive	An authentic GMO can be verified as such. Signature verification protocol returns “ok”	Manufacturer/proxy can successfully run the cryptographic confirmation protocol The existence of the hashes of all the signature transgenes within the GMO is publicly verifiable (e.g., via hybridization)
False positive	The protocol falsely identifies/approves an unauthorized/adulterated GMO (danger of distributing a counterfeit)	Not possible, due to the cryptographic part of the protocol (as a necessary requirement to bring GMOs into circulation) : By virtue of the ZK property, attackers cannot impersonate true manufacturer; hence, cannot sell a counterfeit. The digital part is linked to the physical via signature hashes
True negative	An unauthorized GMO can be confirmed as such. Important that this is done via the physical part of the protocol as the digital part only gives information *about* the object	Physical denial part. Thereby, a GMO is *not* authentic, if at least OFTF: Not the complete set of signature hashes present within the genome (publicly verifiable via PCR, etc.) Verification by competent authorities (who have access to the secret of which clones are valid/dummy), according to the following Identify genetic adulterations (may require WGS) Amplify all valid clones If the illicit genetic alteration is found on a dummy clone, the GMO is a counterfeit
False negative	A genuine GMO is identified as inauthentic	Not possible, due to (1) the correctness/completeness of the digital part (an honest prover can successfully run the protocol), and (2) as long as physical signature components within the genome are stably integrated

Although the method by Mueller et al. (2016) (that part of the cryptographic solution presented herein is based on) focused on GM plants, it can be extended to different GMOs, provided the transgenic cassette can be stably integrated into the host genome. Especially with bacteria, special focus needs to be placed on that, as artificial sequences not integrated into the bacterial genome but onto extra-chromosomal plasmids may be lost (see e.g., Paracchini et al., 2017).

## 6. Conclusion

In contrast to traditional or cryptographic signatures, DNA signatures were not invented in the context of intended intrusions. For practical reasons, the functionality of biologic signatures mostly evolved around balancing sensitivity and specificity. First enhancements to also apprehend some forms of (intended) manipulations were anticipated in Levine (2004) and have become the basis for event-specific GMO detection methods for decades.

The basic idea developed in Levine (2004) incorporates critical components of what is to be expected from a “real” signature. Indeed, the random uptake mechanism of transgenes by *Agrobacterium* creates many gene uptake events of the same transgene into different locations in the host genome. A uniquely identifying event can chosen by selecting both the transgene as well as the accompanying flanking DNA in the host genome. Importantly, due to this mechanism, signatures cannot be reproduced, hence not stolen. It appears that such DNA signatures are at least as reliable as their traditional counterparts. Unfortunately, in the area of modern gene editors, such a signature function alone is not enough. Several risk scenarios have been identified whereby attackers can misuse such signatures via new forms of counterfeiting attacks.

Traditional counterfeiting and blending of high-end products with cheaper material has become a serious problem all around the world. Counterfeit goods have infiltrated most industries from textiles to microchips and pharmaceuticals. And also GMOs! As explained by Berrada et al. (2017), the problem with imitation counterfeits “is that they not only hurt the name of the original and the economy, but because these products are not coming from reliable sources, their quality and efficacy could be compromised.”

Counterfeiting by misusing the prevailing DNA signature format may serve several intents beyond mere cost saving. For instance, the recent B2 contamination event in Europe could have been—or analogously, could be—the result of counterfeiting attacks, whereby the producer is falsely blamed for generating unauthorized GMOs. More serious forms of genetic alterations can be envisioned than the mere overproduction of a compound or trait (that may even be authorized in a different jurisdiction). Counterfeiting may also be based on a malicious intent—to not produce cheaper, but hazardous materials or products, as suggested in Mueller (2019). As long as the alterations are outside the signature sequence that is being used to identify the authenticity of the GMO, such forms of intrusions may analogously remain undetected and give evil-doers a way to circulate GMO weapons masquerading as market GMOs.

These new forms of attacks mandate a re-conceptualization of how DNA signatures need to be realized. Herein, several general recommendations have been made that are based on lessons learned from cryptography, overlaid with practical issues that attackers are facing. Based on these, a specific method is suggested that is able to mitigate the misuse of DNA signatures and the distribution of counterfeits.

DNA-signatures enhanced by ZK-based proofs may be extended to different GM organisms or agents. Of special interest here may be emerging pharmaceutical or medical applications, including medicinal products, gene therapy for biological pacemakers (Farraha et al., 2018) and for the nervous system (Bowers et al., 2011). The full potential of supporting cyberbiosecurity risks via cryptographic and cybersecurity means still remains to be fully fleshed out (see also Diggans and Leproust, 2019; Richardson et al., 2019). DNA signatures are just one example where such interdisciplinary insights can lead to effective and safe security solutions.

## Author Contributions

The author confirms being the sole contributor of this work and has approved it for publication.

### Conflict of Interest Statement

The author declares that the research was conducted in the absence of any commercial or financial relationships that could be construed as a potential conflict of interest.

## Footnotes

^1^Throughout, a hazardous GMO refers to one that intentionally has been manipulated.

## Abbreviations

GM: genetically modified
GMO: GM organism
UGMO: unauthorized GMO
NGS: Next Generation Sequencing
WGS: Whole Genome Sequencing
ZK: Zero Knowledge
TTP: Trusted Third Party.
